# Effects of the Ambient Fine Particulate Matter on Public Awareness of Lung Cancer Risk in China: Evidence from the Internet-Based Big Data Platform

**DOI:** 10.2196/publichealth.8078

**Published:** 2017-10-03

**Authors:** Hongxi Yang, Shu Li, Li Sun, Xinyu Zhang, Jie Hou, Yaogang Wang

**Affiliations:** ^1^ School of Public Health Tianjin Medical University Tianjin China; ^2^ School of Nursing Tianjin Medical University Tianjin China; ^3^ School of Medical English and Health Communication Tianjin Medical University Tianjin China

**Keywords:** lung cancer, risk factors, particulate matter, PM2.5, Baidu Index, information seeking behavior, public awareness, China

## Abstract

**Background:**

In October 2013, the International Agency for Research on Cancer classified the particulate matter from outdoor air pollution as a group 1 carcinogen and declared that particulate matter can cause lung cancer. Fine particular matter (PM_2.5_) pollution is becoming a serious public health concern in urban areas of China. It is essential to emphasize the importance of the public’s awareness and knowledge of modifiable risk factors of lung cancer for prevention.

**Objective:**

The objective of our study was to explore the public’s awareness of the association of PM_2.5_ with lung cancer risk in China by analyzing the relationship between the daily PM_2.5_ concentration and searches for the term “lung cancer” on an Internet big data platform, Baidu.

**Methods:**

We collected daily PM_2.5_ concentration data and daily Baidu Index data in 31 Chinese capital cities from January 1, 2014 to December 31, 2016. We used Spearman correlation analysis to explore correlations between the daily Baidu Index for lung cancer searches and the daily average PM_2.5_ concentration. Granger causality test was used to analyze the causal relationship between the 2 time-series variables.

**Results:**

In 23 of the 31 cities, the pairwise correlation coefficients (Spearman rho) between the daily Baidu Index for lung cancer searches and the daily average PM_2.5_ concentration were positive and statistically significant (*P*<.05). However, the correlation between the daily Baidu Index for lung cancer searches and the daily average PM_2.5_ concentration was poor (all *r*^2^_s_<.1). Results of Granger causality testing illustrated that there was no unidirectional causality from the daily PM_2.5_ concentration to the daily Baidu Index for lung cancer searches, which was statistically significant at the 5% level for each city.

**Conclusions:**

The daily average PM_2.5_ concentration had a weak positive impact on the daily search interest for lung cancer on the Baidu search engine. Well-designed awareness campaigns are needed to enhance the general public’s awareness of the association of PM_2.5_ with lung cancer risk, to lead the public to seek more information about PM_2.5_ and its hazards, and to cope with their environment and its risks appropriately.

## Introduction

Air pollution has become the most severe and worrisome environmental problem and a major threat to public health in China [1-4]. The daily concentration of ambient fine particulate matter <5 μm in diameter (PM_2.5_) and its negative consequences are major public health concerns in China [5-7]. According to the Global Burden of Diseases Study, PM_2.5_ concentration has been the fifth-ranking mortality risk factor, an estimated 4.2 million deaths were attributed to PM_2.5_ around the globe in 2015, and PM_2.5_ contributed to 1.1 million deaths in China in 2015 [8]. In 194 Chinese cities, the total estimated premature deaths and lung cancer deaths attributed to PM_2.5_ pollution were 722,370 and 67,452, respectively, in 2014 and 2015 [5]. The estimated per capita mortality attributable to air pollution by 2050 was projected to be even higher in Chinese megacities [9].

Lung cancer is the most common incident cancer and the leading cause of cancer death in China. New cancer cases and cancer deaths were estimated to be 733,000 and 591,000, respectively, every year [10]. The International Agency for Research on Cancer has classified particulate matter from outdoor air pollution as a group 1 carcinogen that can cause lung cancer [11]. Many studies found that exposure to PM_2.5_ was an important risk factor for lung cancer [12-17]. Increasing the public’s awareness of lung cancer is important for early detection, diagnosis, and intervention of lung cancer.

With the development of a well-established network, the Internet has become a vital channel for the public to access health information. About 63% of cancer patients use the Internet to search for information regarding cancer specifically, and use of the Internet as a source for oncological information is increasing rapidly [5,18,19]. Previous studies have demonstrated that network tools such as Twitter or Google can be used to examine public interest in disease epidemics and to perform disease surveillance, by tracking health-seeking behaviors [20-24].

Baidu is one of the most important Internet big data platforms in China. According to the Chinese Internet Users Search Behavior Study, the Baidu search engine is the most popular among Chinese Internet users, with a priority selection incidence of 93.1% [25]. The Baidu Index stems from search frequencies on the Baidu search engine, and is calculated and displayed on the basis of special keywords by search volume used by netizens on the Baidu search engine. The Baidu Index can serve as a data source for determining the awareness of Internet users on specific topics.

Using the Baidu Index, we examined Chinese public search interest in lung cancer. The goal of this study was to explore public awareness of the association of PM_2.5_ with lung cancer risk by analyzing the relationship between daily PM_2.5_ concentration and daily Baidu Index searches for the term “lung cancer” in China.

## Methods

### Air Pollution Data

We collected air pollution data from the Chinese Air Quality Online Monitoring and Analysis Platform [26], which began air quality monitoring in 2013 for all major Chinese cities. We extracted daily average PM_2.5_ concentration data of 31 Chinese capital cities from January 1, 2014 to December 31, 2016.

### Search Data

The Baidu Index is a useful tool to process and analyze search query data. Its database contains logs of online and mobile phone search query volume submitted from January 2011. The daily Baidu Index is the weighted sum of the search frequency for a keyword based on its daily search volume on the Baidu search engine. The Baidu Index has been proved to be a useful indicator of public interest in and awareness of health-related topics. In this study, we hypothesized that the Baidu Index would offer potential insight into the general population’s awareness of lung cancer. The conceptualized awareness of lung cancer in this study could be considered to be based on the general population’s ability to seek knowledge and information for the disease or pay attention to the disease. We used the Baidu Index to determine the relevance of the search term “lung cancer” as an indicator of the public’s awareness of lung cancer. We collected daily Baidu Index data from the Baidu Index websites [27] for the search term “肺癌” (lung cancer) in Chinese for each of 31 Chinese capital cities from January 1, 2014 to December 31, 2016.

### Baidu Media Data

The Baidu media index is the number of news items containing a specified keyword in their headlines collected by the Baidu news database, sourced from Chinese major websites, including national and local news websites and networks. We collected daily Baidu media index data for the keyword “lung cancer” from the Baidu Index websites [27] from January 1, 2014 to December 31, 2016.

### Statistical Analyses

We calculated descriptive statistics for the 2 variables. These included the mean, standard deviation, median and interquartile range, and the minimum and maximum values of both variables.

We used the Kruskal-Wallis *H* test to examine differences in the daily Baidu Index for lung cancer searches across all cities by month, season, year, and city, separately. We examined the differences in daily PM_2.5_ concentration using the same method.

We used Spearman and Pearson correlation analyses to explore the correlation between the daily Baidu Index for lung cancer searches, daily Baidu media index for lung cancer, and daily average PM_2.5_ concentration, with the statistical significance level set at .01. We calculated Pearson partial correlation coefficients to assess the intercorrelations between the Baidu Index, Baidu media index, and daily PM_2.5_ concentration. Multiple linear regression analysis explored the potential influence of the daily Baidu media index for lung cancer and daily average PM_2.5_ concentration on the daily Baidu Index for lung cancer searches.

Granger causality is a concept of causality derived from the idea that causes cannot occur after effects and that, if one variable is the cause of another, knowing the status of the cause at an earlier point in time can enhance prediction of the effect at a later point in time [28]. We used the Granger causality test to analyze whether there was a causal relationship between the 2 time-series variables. We conducted the Engle-Granger test to examine whether there was co-integration or a long-term association between the 2 time series [29]. In the first step, we used unit root tests to examine whether the time series of the Baidu Index for lung cancer searches and the time series of daily PM_2.5_ concentration were stationary. If the 2 time series were both stationary at the same level, then we estimated the co-integrating regression model using ordinary least squares. We used the daily Baidu Index for lung cancer searches as the dependent variable and the daily average PM_2.5_ concentration as the independent variable. We estimated regression coefficients to assess the effect of daily average PM_2.5_ concentration on the daily Baidu Index for lung cancer searches. In the second step, we used unit root tests to examine whether the residual series of the co-integrating regression model was stationary, which would indicate that the 2 time-series variables were co-integrated, which satisfies the precondition of the Granger causality test. Then, we performed the Granger causality test.

We conducted the descriptive statistics, Kruskal-Wallis H test, and Spearman correlation analysis in IBM SPSS version 19.0 (IBM Corporation), and the Granger causality test using EViews 9 student version (IHS Global Inc).

## Results

### Descriptive Analysis

Table 1 shows the mean and median of the daily Baidu Index for lung cancer searches and daily average PM_2.5_ concentration for each of 31 Chinese cities over the period from January 1, 2014 to December 1, 2016. The mean daily average PM_2.5_ concentration across all cities was 57.37 (SD 47.54) μg/m^3^. For air pollution, Shijiazhuang city in Hebei Province ranked first among the 31 cities, with a mean daily average PM_2.5_ concentration of 104.01 (SD 88.55) μg/m^3^, and Haikou city in Hainan Province ranked last, with a mean daily average PM_2.5_ concentration of 21.64 (SD 15.2) μg/m^3^. In our data, the highest daily average PM_2.5_ concentration was 897.5 μg/m^3^in Shenyang on November 8, 2015, whereas the lowest was 2.5 μg/m^3^in Nanjing on September 16, 2016. The mean daily Baidu Index for lung cancer searches was 180 (SD 83.21) across all cities; that is, there were on average 180 searches daily for the term “lung cancer” from January 1, 2014 to December 31, 2016. The mean of daily Baidu Index for lung cancer searches in Beijing was 408 (SD 69.53) and ranked first among the 31 cities. On May 16, 2016, the daily Baidu Index for lung cancer searches for Beijing peaked at 1185, which was the highest of all Baidu Index data points. The Baidu Index data scale ranged from 0 to 1185, with a median of 73 during the study period. Across all cities, there were significant differences in the daily Baidu Index for lung cancer searches and daily average PM_2.5_ concentration by month, season, year, and city, separately (all *P*<.05).

**Table 1:** Summary statistics of daily average fine particulate matter (PM_2.5_) concentration and daily Baidu Index for the search term “lung cancer” in 31 Chinese capital cities from January 1, 2014 to December 31, 2016.

City	Daily Baidu Index for lung cancer searches		Daily average PM_2.5_ concentration (μg/m^3^)	
	Mean (SD)	Median (25th, 75th percentile)	Mean (SD)	Median (25th, 75th percentile)
Beijing	408 (69.53)	91 (366, 441)	79.23 (68.25)	60.85 (29.9, 106.43)
Changchun	146 (28.52)	74 (139, 162)	58.68 (50.44)	43.25 (28.43, 72.38)
Changsha	195 (31.42)	75.5 (178, 216)	62.8 (39.91)	52.15 (36, 79.2)
Chengdu	277 (58.8)	80 (236,308)	65.9 (44.22)	53.35 (35.53, 82.98)
Chongqing	208 (24.63)	71 (194, 222)	57.24 (34.19)	47.3 (33.9, 68.98)
Fuzhou	165 (28.19)	52 (151, 180)	29.17 (16.14)	26 (18.5, 36.1)
Guangzhou	285 (40.96)	55.5 (258.25, 306.75)	40.78 (21.95)	35.8 (24.4, 51.78)
Guiyang	122 (29.93)	56.5 (87, 143)	40.13 (21.75)	35.9 (24.73, 49.28)
Harbin	168 (24.36)	67 (159, 182)	64.39 (65.96)	40.95 (24.13, 83.48)
Haikou	111 (30.37)	35 (76, 135)	21.64 (15.2)	16.3 (12.3, 26.5)
Hangzhou	250 (42.41)	72 (219.25, 278.75)	55.23 (30.74)	48.9 (33, 70.28)
Hefei	188 (31.58)	85 (171, 209)	67.45 (41.39)	58.2 (41.03, 83.35)
Hohhot	124 (30.98)	74 (91, 146)	42.24 (33.96)	32.05 (19.2, 55.3)
Jinan	196 (29.76)	111 (177, 214)	85.06 (51.35)	73.3 (51.83, 103.28)
Kunming	157 (27.95)	51 (148, 173)	29.79 (13.25)	27.25 (19.9, 37.15)
Lanzhou	117 (32.65)	83 (83, 146)	54.06 (28.25)	46.2 (35.73, 65)
Lhasa	26 (31.4)	57 (0, 57)	25.17 (12.11)	22 (16.5, 30.3)
Nanchang	149 (32.31)	63 (137, 167)	45.21 (30.93)	37.1 (23.5, 57.68)
Nanjing	189 (25.97)	78 (173, 205)	58.49 (38.18)	49.65 (31.03, 76.25)
Nanning	143 (29.23)	57 (133, 161)	41.91 (28.42)	34.15 (22, 53.5)
Shanghai	335 (52.13)	64 (303, 358)	50.17 (32.09)	42.15 (27.2, 65)
Shenyang	171 (25.25)	82 (156, 188)	65.68 (54.92)	49.6 (33.6, 82.43)
Shijiazhuang	176 (32.92)	115 (158, 200)	104.01 (88.55)	80.25 (43.53, 131.1)
Taiyuan	150 (32.5)	88 (137, 171)	64.81 (45.83)	53.15 (32.8, 83.33)
Tianjin	204 (24.9)	90 (186, 220)	74.63 (53.61)	60.6 (37.33, 95.08)
Urumqi	116 (29.92)	87 (82, 139)	66.97 (61.11)	41.65 (27.6, 82.28)
Wuhan	227 (26.54)	87 (212.25, 240)	69.48 (46.95)	58.9 (38.13, 86.28)
Xian	207 (29.64)	87 (192, 223)	68.3 (54.44)	51.35 (35.2, 79.1)
Xining	73 (29.5)	80 (59, 74)	52.63 (24.34)	47.35 (35.73, 65.05)
Yinchuan	82 (30.54)	76 (61, 118)	50.04 (31.12)	40.9 (30.13, 58.38)
Zhengzhou	223 (29.74)	107 (203, 242)	87.02 (61.2)	71.4 (46.8, 108.78)
All cities	180 (83.21)	73 (136, 218)	57.37 (47.54)	44 (27.6, 71.3)

**Figure 1:**
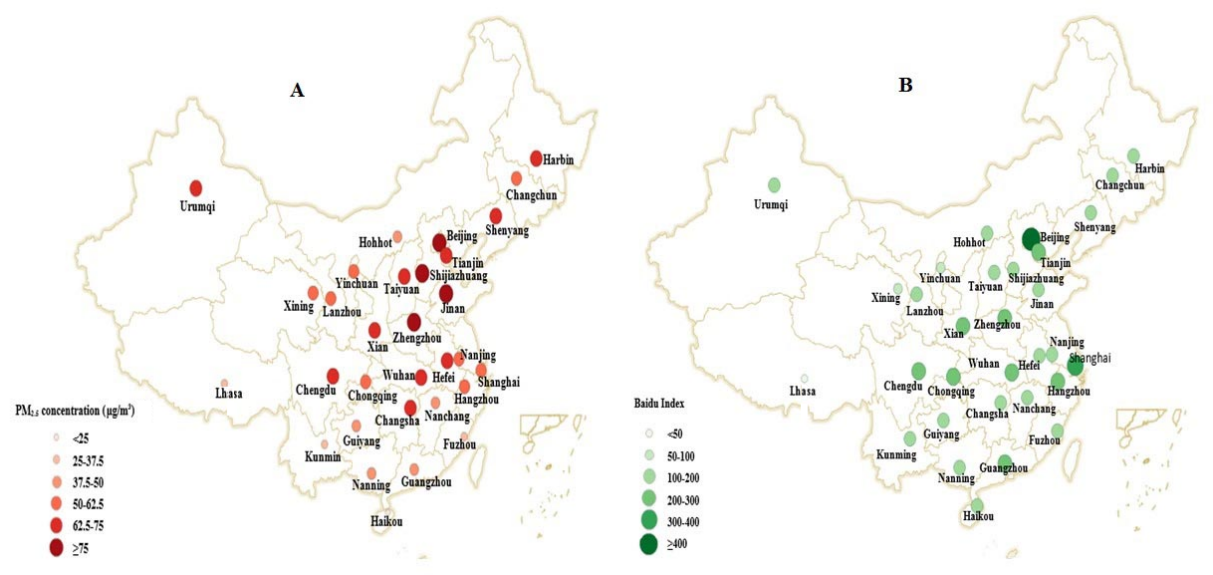
Distribution of (A) mean daily average fine particulate matter (PM_2.5_) concentration and (B) mean daily Baidu Index for the search term “lung cancer” in 31 Chinese capital cities, January 1, 2014 to December 31, 2016.

Compared with 2014, the Baidu Index for lung cancer searches across all cities for 2015 and 2016 decreased by 2% and 5%, respectively. The annual mean daily average PM_2.5_ concentration had decreased slightly from 2014 to 2016. The Baidu media index for lung cancer ranged from 0 to 6523, with a median of 9 (25th, 75th percentile 4, 14) in 2016. The Baidu media index for lung cancer peaked on September 17, 2015. Figure 1 shows the distributions of mean daily average PM_2.5_ concentration and mean daily Baidu Index for lung cancer searches in the 31 Chinese capital cities during the study period.

### Correlation Analysis

Except for Chengdu and Hohhot, the pairwise correlation coefficients (Spearman rho) between the daily Baidu Index for lung cancer searches and daily average PM_2.5_ concentration were positive. Most of the Spearman rank correlation coefficients were statistically significant (*P*<.05). However, the correlations between the daily Baidu Index for lung cancer searches and daily average PM_2.5_ concentration was poor (all *r*^2^_s_<.1) (Table 2). The top 3 correlations were .240 for Hangzhou, .238 for Zhengzhou, and .231 for Hefei (All *P*<.001). For all cities, there was a positive correlation between the daily Baidu Index for lung cancer searches and daily PM_2.5_ concentration (ρ=.247, *P*<.001). Multimedia Appendix 1 shows the results of Pearson correlation analysis and Multimedia Appendix 2 shows the results of multiple linear regression analysis. The correlation between daily PM_2.5_ concentration and the daily Baidu Index for lung cancer searches was more than that between daily PM_2.5_ and daily Baidu media index. When the daily Baidu media index for lung cancer was the control variable, the partial correlation coefficient for each city, between the daily Baidu Index for lung cancer searches and the daily average PM_2.5_ concentration, was almost equal to the Pearson correlation coefficient. The daily Baidu media index for lung cancer had little influence on the relationship between the daily Baidu Index for lung cancer searches and daily average PM_2.5_ concentration according to the partial correlation coefficients. Overall, both the correlation and intercorrelation between the daily Baidu Index for lung cancer searches, daily Baidu media index, and daily PM_2.5_ concentration were poor.

### Granger Causality

We used the augmented Dickey-Fuller unit root test to test the stationarity of the 2 time series. The lag length was determined automatically using the Schwarz information criterion. The series for all cities except Chengdu were stationary at the statistical significance level set at .01, and the series for Chengdu were also stationary at the first difference (Table 3). Since the series for each city were found to be stationary at the same level, therefore, the 2 variables satisfied the precondition of co-integration and were checked for a long-term co-integration relationship.

**Table 2:** Spearman correlation between daily Baidu Index for the search term “lung cancer,” daily Baidu media index, and daily fine particulate matter (PM_2.5_) concentration.

City	Correlation					
	Baidu Index & PM_2.5_		Baidu Index & Baidu media index		Baidu media index & PM_2.5_	
	*r*_s_	*P* value	*r*_s_	*P* value	*r*_s_	*P* value
Beijing	.093^a^	.002	.359^a^	<.001	–.013	.68
Changchun	.060^b^	.048	.167^a^	<.001	.122^a^	<.001
Changsha	.184^a^	<.001	.196^a^	<.001	.047	.12
Chengdu	–.041	.17	.006	.84	.026	.40
Chongqing	.139^a^	<.001	.189^a^	<.001	–.015	.62
Fuzhou	.125^a^	<.001	.191^a^	<.001	.099^a^	.001
Guangzhou	.167^a^	<.001	.303^a^	<.001	.003	.93
Guiyang	.062^b^	.04	.096^a^	.001	.029	.34
Harbin	.219^a^	<.001	.269^a^	<.001	.145^a^	<.001
Haikou	.091^a^	.002	.164^a^	<.001	–.018	.55
Hangzhou	.240^a^	<.001	.253^a^	<.001	.133^a^	<.001
Hefei	.231^a^	<.001	.225^a^	<.001	.098^a^	.001
Hohhot	.036	.23	.188^a^	<.001	.028	.36
Jinan	.149^a^	<.001	.164^a^	<.001	.052	.09
Kunming	.024	.42	.081^a^	.007	.031	.30
Lanzhou	.057	.06	.149^a^	<.001	.046	.13
Lhasa	.001	.98	.039	.19	.123^a^	<.001
Nanchang	.118^a^	<.001	.228^a^	<.001	.047	.12
Nanjing	.220^a^	<.001	.232^a^	<.001	.125^a^	<.001
Nanning	.039	.19	.165^a^	<.001	–.005	.87
Shanghai	.050	.10	.269^a^	<.001	.115^a^	<.001
Shenyang	.100^a^	.001	.276^a^	<.001	.103^a^	.001
Shijiazhuang	.204^a^	<.001	.179^a^	<.001	.030	.33
Taiyuan	.088^a^	.003	.218^a^	<.001	.027	.38
Tianjin	.085^a^	.005	.290^a^	<.001	.037	.22
Urumqi	.153^a^	<.001	.115^a^	<.001	.058	.06
Wuhan	.154^a^	<.001	.201^a^	<.001	.055	.07
Xian	.111^a^	<.001	.253^a^	<.001	.035	.25
Xining	.081^a^	.007	.105^a^	.001	.109^a^	<.001
Yinchuan	–.012	.68	.102^a^	.001	.012	.69
Zhengzhou	.238^a^	<.001	.277^a^	<.001	.085^a^	.005

^a^Correlation is significant at the .01 level (2-tailed).

^b^Correlation is significant at the .05 level (2-tailed).

**Table 3:** Results of unit root tests for the time series of daily average fine particulate matter (PM_2.5_) concentration and daily Baidu Index for the search term “lung cancer.”

City	Unit root test for time series of daily Baidu Index for lung cancer searches			Unit root test for time series of daily PM_2.5_ concentration			Result^a^
	ADF^b^	1% Level	*P* value	ADF	1% Level	*P* value	
Beijing	–5.23	–3.44	<.001	–18.44	–3.44	<.001	Stationarity
Changchun	–7.83	–3.44	<.001	–7.32	–3.44	<.001	Stationarity
Chengdu	–23.41	–3.44	<.001	–17.18	–3.44	<.001	Stationarity
Chongqing	–5.70	–3.44	<.001	–10.18	–3.44	<.001	Stationarity
Changsha	–5.25	–3.44	<.001	–9.76	–3.44	<.001	Stationarity
Fuzhou	–5.00	–3.44	<.001	–8.88	–3.44	<.001	Stationarity
Guiyang	–29.62	–3.44	<.001	–8.78	–3.44	<.001	Stationarity
Guizhou	–6.01	–3.44	<.001	–13.39	–3.44	<.001	Stationarity
Harbin	–7.21	–3.44	<.001	–6.49	–3.44	<.001	Stationarity
Hefei	–4.50	–3.44	<.001	–7.13	–3.44	<.001	Stationarity
Hohhot	–27.24	–3.44	<.001	–4.58	–3.44	<.001	Stationarity
Haikou	–30.13	–3.44	<.001	–10.92	–3.44	<.001	Stationarity
Hangzhou	–3.79	–3.44	<.001	–4.53	–3.44	<.001	Stationarity
Jinan	–5.10	–3.44	<.001	–15.65	–3.44	<.001	Stationarity
Kunming	–7.64	–3.44	<.001	–9.28	–3.44	<.001	Stationarity
Lhasa	–4.33	–2.57	<.001	–4.24	–3.44	<.001	Stationarity
Lanzhou	–28.38	–3.44	<.001	–5.47	–3.44	<.001	Stationarity
Nanchang	–7.17	–3.44	<.001	–10.42	–3.44	<.001	Stationarity
Nanjing	–5.60	–3.44	<.001	–6.29	–3.44	<.001	Stationarity
Nanning	–7.48	–3.44	<.001	–7.62	–3.44	<.001	Stationarity
Shanghai	–5.68	–3.44	<.001	–18.64	–3.44	<.001	Stationarity
Shijiazhuang	–4.58	–3.44	<.001	–4.71	–3.44	<.001	Stationarity
Shenyang	–5.23	–3.44	<.001	–12.08	–3.44	<.001	Stationarity
Tianjin	–5.84	–3.44	<.001	–6.99	–3.44	<.001	Stationarity
Taiyuan	–5.80	–3.44	<.001	–4.69	–3.44	<.001	Stationarity
Wuhan	–5.47	–3.44	<.001	–4.38	–3.44	<.001	Stationarity
Xian	–5.20	–3.44	<.001	–4.31	–3.44	<.001	Stationarity
Xining	–30.74	–3.44	<.001	–5.49	–3.44	<.001	Stationarity
Yinchuan	–19.20	–3.44	<.001	–10.21	–3.44	<.001	Stationarity
Zhengzhou	–5.16	–3.44	<.001	–5.44	–3.44	<.001	Stationarity

^a^Time series of daily average PM_2.5_ concentration and of daily Baidu Index for lung cancer were stationary at the same level.

^b^ADF: augmented Dickey-Fuller unit root test.

**Figure 2:**
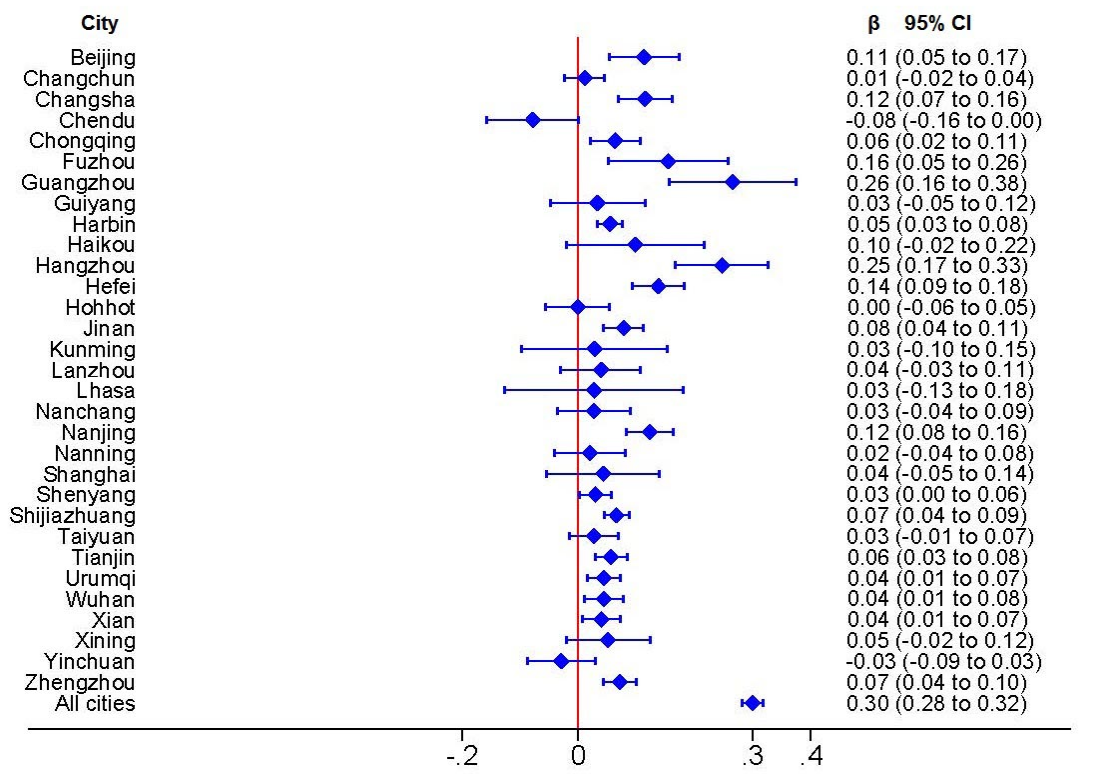
Estimate of regression coefficient (β) with 95% CI.

For 17 of the 31 Chinese capital cities, regression analysis revealed that the positive effects of daily average PM_2.5_ concentration on the daily Baidu Index for lung cancer searches were statistically significant (Figure 2). We observed the strongest relationship in Guangzhou as indicated by a regression coefficient of 0.26 (95% CI 0.16-0.38). The effect of daily average PM_2.5_ concentration was negative in Chengdu and Yinchuan, but was not statistically significant, with a regression coefficient of –0.08 (95% CI –0.16 to 0) for Chengdu and –0.03 (95% CI –0.09 to 0.03) for Yinchuan. For all cities, the regression coefficient was 0.30 (95% CI 0.28-0.32). Overall, the relationship between daily average PM_2.5_ concentration and the daily Baidu Index for lung cancer searches was modest.

The result of the panel co-integration (Engle-Granger) test indicated the existence of co-integration between variables for each city at the 1% significance level (Table 4). The co-integration test revealed the existence of a long-term relationship between variables but did not indicate the direction of the causal relationship. The results of the Granger causality test suggested the absence of a unidirectional causality from PM_2.5_ to the Baidu Index, which was statistically significant at the 5% level for all cities (Table 5).

**Table 4:** Results of co-integration test of the 2 time series of daily average fine particulate matter (PM_2.5_) concentration and daily Baidu Index for the search term “lung cancer.”

City	Unit root test for the residual series					Result^a^
	ADF^b^	1% Level	5% Level	10% Level	*P* value	
Beijing	–5.26	–2.57	–1.94	–1.62	<.001	Co-integration
Changchun	–7.84	–3.44	–2.86	–2.57	<.001	Co-integration
Chengdu	–23.32	–3.44	–2.86	–2.57	<.001	Co-integration
Chongqing	–5.89	–3.44	–2.86	–2.57	<.001	Co-integration
Changsha	–5.52	–3.44	–2.86	–2.57	<.001	Co-integration
Fuzhou	–5.08	–3.44	–2.86	–2.57	<.001	Co-integration
Guiyang	–29.63	–3.44	–2.86	–2.57	<.001	Co-integration
Guizhou	–6.18	–3.44	–2.86	–2.57	<.001	Co-integration
Harbin	–7.53	–3.44	–2.86	–2.57	<.001	Co-integration
Hefei	–4.74	–3.44	–2.86	–2.57	<.001	Co-integration
Hohhot	–27.24	–3.44	–2.86	–2.57	<.001	Co-integration
Haikou	–30.22	–3.44	–2.86	–2.57	<.001	Co-integration
Hangzhou	–4.06	–3.44	–2.86	–2.57	<.001	Co-integration
Jinan	–5.24	–3.44	–2.86	–2.57	<.001	Co-integration
Kunming	–7.65	–3.44	–2.86	–2.57	<.001	Co-integration
Lhasa	–20.05	–3.44	–2.86	–2.57	<.001	Co-integration
Lanzhou	–28.46	–3.44	–2.86	–2.57	<.001	Co-integration
Nanchang	–7.22	–3.44	–2.86	–2.57	<.001	Co-integration
Nanjing	–5.78	–3.44	–2.86	–2.57	<.001	Co-integration
Nanning	–7.48	–3.44	–2.86	–2.57	<.001	Co-integration
Shanghai	–5.71	–3.44	–2.86	–2.57	<.001	Co-integration
Shijiazhuang	–4.85	–3.44	–2.86	–2.57	<.001	Co-integration
Shenyang	–5.35	–3.44	–2.86	–2.57	<.001	Co-integration
Tianjin	–5.95	–3.44	–2.86	–2.57	<.001	Co-integration
Taiyuan	–5.83	–3.44	–2.86	–2.57	<.001	Co-integration
Wuhan	–5.51	–3.44	–2.86	–2.57	<.001	Co-integration
Xian	–5.21	–3.44	–2.86	–2.57	<.001	Co-integration
Xining	–30.80	–3.44	–2.86	–2.57	<.001	Co-integration
Yinchuan	–19.20	–3.44	–2.86	–2.57	<.001	Co-integration
Zhengzhou	–5.44	–3.44	–2.86	–2.57	<.001	Co-integration

^a^Time series of daily average PM_2.5_ concentration and of daily Baidu Index for lung cancer were co-integrated.

^b^ADF: augmented Dickey-Fuller unit root test.

**Table 5:** Results of Granger causality test of the causal relationship between daily average fine particulate matter (PM_2.5_) concentration and daily Baidu Index for the search term “lung cancer.”

City	Null hypothesis^a^					
	Daily average PM_2.5_ concentration does not Granger cause daily Baidu Index for lung cancer searches			Daily Baidu Index for lung cancer searches does not Granger cause daily average PM_2.5_ concentration		
	*F* statistic	*df*	*P* value	*F* statistic	*df*	*P* value
Beijing	1.25	15, 1066	.23	0.66	15, 1066	.81
Changchun	1.62	5, 1086	.15	0.30	5, 1086	.91
Chengdu	0.17	5, 1086	.97	1.39	5, 1086	.22
Chongqing	1.32	6, 1084	.24	1.41	6, 1084	.20
Changsha	1.30	6, 1084	.25	1.14	6, 1084	.33
Fuzhou	0.93	6, 1079	.47	1.21	6, 1079	.29
Guiyang	0.19	1, 1090	.66	0.36	1, 1090	.54
Guizhou	1.54	5, 1086	.17	1.84	5, 1086	.10
Harbin	1.14	6, 1084	.33	2.78	6, 1084	.01
Hefei	1.94	6, 1084	.07	2.62	6, 1084	.01
Hohhot	1.72	1, 1090	.19	0.15	1, 1090	.69
Haikou	1.69	1,1090	.19	1.44	1,1090	.22
Hangzhou	0.86	6, 1084	.52	1.86	6, 1084	.08
Jinan	1.57	6, 1084	.15	1.42	6, 1084	.20
Kunming	1.13	6, 1084	.34	0.88	6, 1084	.50
Lhasa	0.81	1, 1090	.37	0.14	1, 1090	.70
Lanzhou	1.97	1, 1090	.16	2.91	1, 1090	.08
Nanchang	1.28	6, 1084	.26	1.45	6, 1084	.19
Nanjing	1.49	6, 1084	.18	2.05	6, 1084	.05
Nanning	0.62	5, 1086	.68	1.82	5, 1086	.10
Shanghai	0.59	8, 1080	.79	0.65	8, 1080	.73
Shijiazhuang	0.52	6, 1084	.79	1.62	6, 1084	.13
Shenyang	0.39	6, 1078	.88	0.88	6, 1078	.50
Tianjin	0.89	5, 1086	.48	0.90	5, 1086	.47
Taiyuan	2.42	7, 1082	.02	0.66	7, 1082	.7
Wuhan	0.95	6, 1084	.45	1.09	6, 1084	.36
Xian	1.35	7, 1082	.22	0.72	7, 1082	.64
Xining	3.40	1, 1090	.06	0.17	1, 1090	.67
Yinchuan	0.24	1, 1090	.61	0.00	1, 1090	.99
Zhengzhou	1.71	6, 1084	.11	1.28	6, 1084	.26

^a^Null hypothesis is rejected when *P*<.01.

## Discussion

### Principal Results

Our analysis showed a slightly positive correlation between daily average PM_2.5_ concentration and the daily Baidu Index for the search term “lung cancer” in most of the 31 cities. The result of the regression analysis also showed that daily average PM_2.5_ concentration had a weak impact on the daily Baidu Index for lung cancer searches. The Granger causality test indicated that there was no causal relationship between daily average PM_2.5_ concentration and the daily Baidu Index for lung cancer searches.

Some studies have assessed the association between PM_2.5_ and subsequent risks of lung cancer incidence and mortality, suggesting that PM_2.5_ could be a risk factor for lung cancer. Therefore, the mass media in China often remind people to use the necessary protection at a high concentration of PM_2.5_. The public’s search interest in lung cancer reflects their concern about this disease. In China, the general population can easily get daily information about the PM_2.5_ concentration through the government’s official website, the news media, and many weather forecast mobile phone apps. However, little is known about whether the reported daily information about PM_2.5_ concentration significantly stimulates the public’s interest in lung cancer in China. Google Trends and the Baidu Index have proved to be useful indicators of public interest in and attention to health-related topics [30-32]. In our study, we hypothesized that the Baidu Index would offer potential insight into the general population’s interest in lung cancer as a reflection of the daily PM_2.5_ concentration.

Wang et al [30] investigated the value of Chinese social media for monitoring air quality trends and related public perceptions and response; they found that media data contain rich details, including perceptions, behaviors, and self-reported health effects, which provides a theoretical basis for our research. In our study, we extracted real search data from the Baidu search engine and we examined the relationship between the reported daily PM_2.5_ concentration data and the search data for the specific search term “lung cancer” to test our hypothesis.

In 2013, the European Study of Cohorts for Air Pollution Effects reported that each 5 μg/m^3^increase of PM_2.5_ was statistically significantly associated with a hazard ratio for lung cancer of 1.18 (95% CI 0.96-1.46) [12]. Many studies had indicated the PM_2.5_ could cause lung cancer, and there are still many ongoing studies on the relationship between PM_2.5_ and lung cancer. However, it’s still unknown whether the association between PM_2.5_ and lung cancer risk has been recognized by the general public. In addition to traditional methods such as surveys and interviews, we can use Internet-based data to investigate the existing perception and augment health-related data. We therefore used Baidu Index data to measure the public’s awareness of the association of PM_2.5_ with an increased risk of lung cancer.

Our result showed that the daily average PM_2.5_ concentration had a modest impact on the daily Baidu Index for lung cancer searches, but there was still substantial uncertainty about the association. First, the effect of daily average PM_2.5_ concentration on the public’s awareness of its health hazards might be marginal. People may not be concerned much about lung cancer risks until serious health hazards of PM_2.5_ emerge. However, online searches for lung cancer may decline when the significance of PM_2.5_ has become widely recognized. Similarly, the initial panic over lung cancer caused by some events might increase searches for the term “lung cancer” during the first few days, which may drop after the initial panic; such possibilities may have biased our results. Second, lung cancer is a chronic disease with a slow onset, and exposure to PM_2.5_ is more detrimental to lung cancer risk in the long term. The daily average PM_2.5_ concentration had a relatively long, slow impact on the search rate for lung cancer, indicating a possible long time lag in the relationship. Third, lack of awareness that PM_2.5_ can increase the risk of lung cancer might have an important effect on the association between PM_2.5_ and the Baidu Index for lung cancer searches.

China is a vast and diverse country, with a population of more than 1.3 billion people. The effect of PM_2.5_ on the Baidu Index for lung cancer searches might also depend on demographic and socioeconomic conditions, and differences in health literacy among residents in different cities. For the city Shijiazhuang, the daily average PM_2.5_ concentration was highest, but the Baidu Index for lung cancer searches was significantly lower than for some developed cities, such as Beijing, Fuzhou, and Guangzhou. People in the densely populated and economically developed cities in east China have higher health awareness, have better access to the Internet, and more frequently search for health information than do people in sparsely populated and developing cities. The daily average PM_2.5_ concentration in Lhasa was similar to that in Haikou, but the Baidu Index for lung cancer searches in these 2 cities was notably different. In our data, the mean daily average PM_2.5_ concentration across all cities was 53.47 (SD 47.54) μg/m^3^, which is more than the World Health Organization standard of 25 μg/m^3^[33]. Although air quality has been improving in recent years, PM_2.5_ pollution in wintertime is worsening, especially in northern China. PM_2.5_ pollution is an emerging problem that threatens public health, especially in Chinese megacity clusters [34]. People in most of the 31 cities in China that we studied had serious health problems attributed to PM_2.5_. Therefore, the health effects of PM_2.5_ on a local scale for each city need be taken seriously. Local authorities should make a greater effort to improve the air quality and the eHealth literacy in their cities. Online health information should be made more accessible to the public, especially in economically underdeveloped areas.

November is Lung Cancer Awareness Month internationally, and November 18 is Lung Cancer Day, which aim to raise lung cancer awareness among the public. In this study, we found a significant difference in the Baidu media index of lung cancer among different months by Kruskal-Wallis *H* test. The mean rank of the Baidu media index was highest in November (Multimedia Appendix 3); however, it was not highest for any of the 31 cities individually in November. The influence of the Lung Cancer Awareness Month campaign on public interest in lung cancer searches in China was below our expectations. According to the analyses of correlation between the daily Baidu Index, the daily Baidu media index, and the daily PM_2.5_ concentration, both the correlation and intercorrelation between these variables were poor, and the daily Baidu media index had little impact on the correlation between the daily Baidu Index and the daily PM_2.5_ concentration. This suggests that the reported daily PM_2.5_ concentration might have little impact on increasing either the public’s or the media’s attention to lung cancer in China.

Contrary to our expectations, the daily average PM_2.5_ concentration did not notably enhance the public’s awareness of lung cancer. Lung cancer is one of the most prevalent and deadliest cancers. An increase of 10 μg/m^3^of PM_2.5_ could result in up to a 22% increase in lung cancer prevalence [12,35]. It is vital to emphasize the importance of the public’s awareness and knowledge of modifiable risk factors of lung cancer for prevention. Lack of awareness of the risk for lung cancer due to PM_2.5_ might have deleterious consequences for the public, in consideration of lifestyle modification and risk factor avoidance, and might limit the public’s participation in lung cancer prevention or the avoidance of PM_2.5_. Enhancing this awareness might raise self-protective avoidance of lung cancer risk factors. Ngo et al indicated that awareness of the connection between air pollution and its negative health effects can help the public improve their understanding of air pollution and develop responses to it. This awareness could also lead the public to seek more information about air pollution and its hazards, and to cope with their environment and its risks [36]. According to the 39th China statistical report on Internet development, 195 million people used the Internet for health care, with an annual growth rate of 28%, and the number of queries for health information was up 10.8% in 2016 [37]. The Internet can be treated as a sensor of perceptions, behaviors, and self-reported health effects [30]. This advantage of the Internet and social media should be used fully to increase public awareness of the association of PM_2.5_ with lung cancer risk. At the same time, monitoring the public response to the health hazards of PM_2.5_ is necessary to avoid causing social panics. Limited awareness about cancer can hamper primary prevention and the early detection of cancer, as can lack of awareness about the association of PM_2.5_ with lung cancer risk. Cancer awareness campaigns can effectively stimulate the response and online activities of the general public, and can improve knowledge and awareness of cancer [31,32]. Awareness campaigns are needed to increase public knowledge of the lung cancer risk of PM_2.5_ and should be designed to improve knowledge of lung cancer and promote actively taking effective measures to reduce exposure to PM_2.5_ on hazy days.

### Strengths and Limitations

The strength of this study is that it is the first, to our knowledge, to explore the relationship between daily average PM_2.5_ concentration and the daily Baidu Index for the search term “lung cancer” across 31 cities in China.

There are some limitations to this study. We collected the Internet search data from a single search engine, Baidu. Baidu is the most commonly used search engine in China. The Baidu Index provides absolute search data by cities and can be used to perform a direct comparative analysis among cities. We used only the term “lung cancer,” which might have limited the search data. It was also not possible to identify the type of Internet user or which stakeholders were responsible for the search activity. Search engine search term trends might be affected by factors such as public panic [38]. Some people might have searched the term “lung cancer” for other purposes. That the search data are affected by such random factors is an unavoidable limitation in studies using search engine data. We, and many other scholars, are committed to solving this problem and are seeking ways to identify and reduce biases that are embedded in search engine data. This study was also limited by the study areas. We only focused on 31 cities, so the results cannot be extrapolated to other cities and rural areas. It was beyond the scope of our work to explore the relation between PM_2.5_ and online searches for information on other diseases or the relation between online searches for lung cancer and other risk factors.

### Conclusion

Daily average PM_2.5_ concentration has a weak positive impact on Internet searches on the term “lung cancer.” Well-designed awareness campaigns are needed to improve general public awareness of the association of PM_2.5_ with lung cancer risk, to lead the public to seek more information about PM_2.5_ and its hazards, and to cope with their environment and its risks appropriately.

## Appendix

Multimedia Appendix 1 Results of Pearson correlation analysis.

Multimedia Appendix 2 Results of multiple linear regression analysis.

Multimedia Appendix 3 Mean rank of the daily Baidu Index for the term “lung cancer," daily Baidu media index, and daily PM_2.5_ concentration for different months from 2014 to 2016.

## Abbreviations

PM _2.5_: ambient fine particulate matter <5 μm in diameter
