# Supplementary material for: Effects of the Ambient Fine Particulate Matter on Public Awareness of Lung Cancer Risk in China: Evidence from the Internet-Based Big Data Platform
Source: JMIR Public Health Surveill. 2017 Oct 3;3(4):e64. doi: 10.2196/publichealth.8078 (PMC5645640; doi:10.2196/publichealth.8078)
Supplement: Multimedia Appendix 1 [file publichealth_v3i4e64_app1.pdf]

	B&P		B&M		P&M		B&P	
	r_s	*P*	r_s	*P*	r_s	*P*	r_s	*P*
Beijing	.111[a]	<0.001	0.026	0.398	0.023	0.448	0.111	<0.001
Changchun	0.019	0.539	0.031	0.305	-0.003	0.929	0.019	0.537
Changsha	.147[a]	<0.001	0.024	0.432	-0.013	0.675	0.147	<0.001
Chengdu	-0.059	0.051	0.015	0.611	-0.033	0.270	-0.059	0.053
Chongqing	.088[a]	0.004	0.046	0.126	-0.023	0.451	0.089	0.003
Fuzhou	.089[a]	0.003	0.032	0.292	-0.006	0.833	0.089	0.003
Guangzhou	.142[a]	<0.001	0.052	0.086	0.005	0.874	0.142	<0.001
Guiyang	0.024	0.426	0.044	0.148	0.023	0.441	0.023	0.445
Harbin	.148[a]	<0.001	0.027	0.369	-0.007	0.826	0.148	<0.001
Haikou	0.049	0.104	-0.024	0.436	-0.022	0.465	0.049	0.108
Hangzhou	.179[a]	<0.001	0.017	0.569	-0.004	0.893	0.179	<0.001
Hefei	.181[a]	<0.001	0.016	0.588	-0.015	0.624	0.181	<0.001
Hohhot	-0.002	0.958	0.037	0.217	0.016	0.591	-0.002	0.942
Jinan	.134[a]	<0.001	0.033	0.274	-0.006	0.837	0.134	<0.001
Kunming	0.013	0.659	0.042	0.162	-0.009	0.755	0.014	0.649
Lanzhou	0.033	0.269	0.053	0.078	-0.014	0.636	0.034	0.258
Lasa	0.010	0.733	-0.013	0.678	-0.020	0.499	0.010	0.740
Nanchang	0.026	0.397	0.013	0.657	-0.025	0.411	0.026	0.391
Nanjing	.181[a]	<0.001	<0.001	0.993	-0.023	0.445	0.181	<0.001
Nanning	0.019	0.529	0.033	0.281	-0.009	0.774	0.019	0.523
Shanghai	0.026	0.385	0.025	0.415	-0.013	0.659	0.027	0.379
Shenyang	.064[b]	0.034	0.016	0.606	-0.002	0.952	0.064	0.034
Shijiazhuang	.178[a]	<0.001	0.015	0.627	-0.007	0.808	0.178	<0.001
Taiyuan	0.038	0.203	0.046	0.124	0.017	0.573	0.038	0.212
Tianjin	.121[a]	<0.001	0.013	0.662	-0.006	0.846	0.121	<0.001
Urumqi	.089[a]	0.003	0.021	0.482	-0.024	0.432	0.089	0.003
Wuhan	.077[b]	0.011	0.014	0.638	-0.020	0.499	0.077	0.010
Xian	.073[b]	0.016	0.030	0.321	0.001	0.979	0.073	0.016
Xining	0.042	0.160	0.001	0.969	-0.023	0.449	0.043	0.160
Yinchuan	-0.029	0.335	-0.027	0.367	0.014	0.654	-0.029	0.342
Zhengzhou	.146[a]	<0.001	0.031	0.310	-0.001	0.986	0.146	<0.001

Table 1. The Pearson correlation between the daily Baidu Index for the term "lung cancer"(B), the daily Baidu media index of lung cancer (M) and the daily $PM_{2.5}$ concentrations(P)

Note: [a] Correlation is significant at the 0.01 level (2-tailed).
[b] Correlation is significant at the 0.05 level (2-tailed).
