# Supplementary material for: Effects of the Ambient Fine Particulate Matter on Public Awareness of Lung Cancer Risk in China: Evidence from the Internet-Based Big Data Platform
Source: JMIR Public Health Surveill. 2017 Oct 3;3(4):e64. doi: 10.2196/publichealth.8078 (PMC5645640; doi:10.2196/publichealth.8078)
Supplement: Multimedia Appendix 2 [file publichealth_v3i4e64_app2.pdf]

Table 1. The results of the multiple liner regression analysis (Dependent variable Y:the daily Baidu index for the term “lung cancer”, Independent variable X and M: the daily Baidu media index and the daily PM2.5 concentration)

City	Model	B	SE	Beta	t	Sig.	Correlations		
							Zero-order	Partial	Part
Beijing	(Constant)	398.606	3.207						
	X	0.113	0.031	0.111	3.68	0	0.111	0.111	0.111
	M	0.008	0.01	0.023	0.766	0.444	0.026	0.023	0.023
Changchun	(Constant)	145.449	1.327		109.616	0			
	X	0.011	0.017	0.019	0.617	0.537	0.019	0.019	0.019
	M	0.004	0.004	0.031	1.027	0.305	0.031	0.031	0.031
Changsha	(Constant)	187.754	1.757		106.841	0			
	X	0.116	0.024	0.147	4.914	0	0.147	0.147	0.147
	M	0.004	0.005	0.026	0.856	0.392	0.024	0.026	0.026
Chengdu	(Constant)	282.406	3.2		88.24	0			
	X	-0.078	0.04	-0.059	-1.939	0.053	-0.059	-0.059	-0.059
	M	0.004	0.009	0.013	0.445	0.656	0.015	0.013	0.013
Chongqing	(Constant)	204.661	1.45		141.128	0			
	X	0.064	0.022	0.089	2.948	0.003	0.088	0.089	0.089
	M	0.006	0.004	0.048	1.602	0.11	0.046	0.048	0.048
Fuzhou	(Constant)	159.915	1.761		90.818	0			
	X	0.155	0.053	0.089	2.942	0.003	0.089	0.089	0.089
	M	0.004	0.004	0.033	1.077	0.282	0.032	0.033	0.033
Guangzhou	(Constant)	274.074	2.588		105.91	0			
	X	0.265	0.056	0.142	4.744	0	0.142	0.142	0.142
	M	0.01	0.006	0.051	1.713	0.087	0.052	0.052	0.051
Guiyang	(Constant)	120.454	1.898		63.449	0			
	X	0.032	0.042	0.023	0.764	0.445	0.024	0.023	0.023

City	Model	B	SE	Beta	t	Sig.	Correlations		
							Zero-order	Partial	Part
	M	0.006	0.004	0.043	1.431	0.153	0.044	0.043	0.043
Haerbin	(Constant)	164.863	1.022		161.257	0			
	X	0.055	0.011	0.148	4.941	0	0.148	0.148	0.148
	M	0.003	0.004	0.028	0.941	0.347	0.027	0.028	0.028
Haikou	(Constant)	109.363	1.602		68.277	0			
	X	0.097	0.06	0.049	1.611	0.108	0.049	0.049	0.049
	M	-0.003	0.005	-0.022	-0.744	0.457	-0.024	-0.022	-0.022
Hangzhou	(Constant)	236.41	2.6		90.917	0			
	X	0.247	0.041	0.179	6.006	0	0.179	0.179	0.179
	M	0.004	0.006	0.018	0.603	0.547	0.017	0.018	0.018
Hefei	(Constant)	178.222	1.801		98.965	0			
	X	0.138	0.023	0.181	6.086	0	0.181	0.181	0.181
	M	0.003	0.005	0.019	0.641	0.522	0.016	0.019	0.019
Huhehot	(Constant)	124.203	1.497		82.953	0			
	X	-0.002	0.028	-0.002	-0.073	0.942	-0.002	-0.002	-0.002
	M	0.006	0.005	0.037	1.237	0.216	0.037	0.037	0.037
Jinan	(Constant)	189.677	1.729		109.706	0			
	X	0.078	0.017	0.134	4.471	0	0.134	0.134	0.134
	M	0.005	0.004	0.034	1.131	0.258	0.033	0.034	0.034
Kunming	(Constant)	155.677	2.081		74.792	0			
	X	0.029	0.064	0.014	0.455	0.649	0.013	0.014	0.014
	M	0.006	0.004	0.042	1.404	0.161	0.042	0.042	0.042
Lanzhou	(Constant)	114.795	2.133		53.822	0			
	X	0.04	0.035	0.034	1.133	0.258	0.033	0.034	0.034
	M	0.009	0.005	0.054	1.78	0.075	0.053	0.054	0.054

City	Model	B	SE	Beta	t	Sig.	Correlations		
							Zero-order	Partial	Part
Lasa	(Constant)	25.387	2.196		11.563	0			
	X	0.026	0.078	0.01	0.332	0.74	0.01	0.01	0.01
	M	-0.002	0.005	-0.012	-0.409	0.683	-0.013	-0.012	-0.012
Nanchang	(Constant)	147.336	1.737		84.835	0			
	X	0.027	0.032	0.026	0.859	0.391	0.026	0.026	0.026
	M	0.002	0.005	0.014	0.465	0.642	0.013	0.014	0.014
Nanjing	(Constant)	182.034	1.418		128.333	0			
	X	0.123	0.02	0.181	6.088	0	0.181	0.181	0.181
	M	0.001	0.004	0.004	0.149	0.881	0	0.005	0.004
Nanning	(Constant)	142.311	1.579		90.152	0			
	X	0.02	0.031	0.019	0.639	0.523	0.019	0.019	0.019
	M	0.005	0.004	0.033	1.083	0.279	0.033	0.033	0.033
Shanghai	(Constant)	333.046	2.933		113.549	0			
	X	0.043	0.049	0.027	0.88	0.379	0.026	0.027	0.027
	M	0.006	0.008	0.025	0.826	0.409	0.025	0.025	0.025
Shenyang	(Constant)	169.278	1.195		141.654	0			
	X	0.03	0.014	0.064	2.121	0.034	0.064	0.064	0.064
	M	0.002	0.004	0.016	0.521	0.602	0.016	0.016	0.016
Shijiazhaung	(Constant)	168.925	1.517		111.375	0			
	X	0.066	0.011	0.178	5.986	0	0.178	0.178	0.178
	M	0.003	0.005	0.016	0.537	0.591	0.015	0.016	0.016
Taiyuan	(Constant)	147.65	1.702		86.758	0			
	X	0.027	0.021	0.038	1.248	0.212	0.038	0.038	0.038
	M	0.007	0.005	0.046	1.516	0.13	0.046	0.046	0.046
Tianjin	(Constant)	199.702	1.285		155.398	0			

City	Model	B	SE	Beta	t	Sig.	Correlations		
							Zero-order	Partial	Part
	X	0.056	0.014	0.121	4.041	0	0.121	0.121	0.121
	M	0.002	0.004	0.014	0.464	0.643	0.013	0.014	0.014
Urumuqi	(Constant)	112.829	1.343		83.989	0			
	X	0.044	0.015	0.089	2.969	0.003	0.089	0.089	0.089
	M	0.003	0.004	0.023	0.776	0.438	0.021	0.023	0.023
Wuhan	(Constant)	223.796	1.435		155.977	0			
	X	0.044	0.017	0.077	2.564	0.01	0.077	0.077	0.077
	M	0.002	0.004	0.016	0.524	0.6	0.014	0.016	0.016
Xian	(Constant)	204.587	1.438		142.304	0			
	X	0.04	0.016	0.073	2.414	0.016	0.073	0.073	0.073
	M	0.004	0.004	0.03	0.992	0.321	0.03	0.03	0.03
Xining	(Constant)	70.686	2.129		33.209	0			
	X	0.052	0.037	0.043	1.406	0.16	0.042	0.043	0.043
	M	0	0.004	0.002	0.071	0.943	0.001	0.002	0.002
Yinchuan	(Constant)	83.055	1.75		47.453	0			
	X	-0.028	0.03	-0.029	-0.952	0.342	-0.029	-0.029	-0.029
	M	-0.004	0.005	-0.027	-0.89	0.373	-0.027	-0.027	-0.027
Zhengzhou	(Constant)	216.278	1.55		139.538	0			
	X	0.071	0.015	0.146	4.889	0	0.146	0.146	0.146
	M	0.004	0.004	0.031	1.028	0.304	0.031	0.031	0.031
