# Supplementary material for: Effects of the Ambient Fine Particulate Matter on Public Awareness of Lung Cancer Risk in China: Evidence from the Internet-Based Big Data Platform
Source: JMIR Public Health Surveill. 2017 Oct 3;3(4):e64. doi: 10.2196/publichealth.8078 (PMC5645640; doi:10.2196/publichealth.8078)
Supplement: Multimedia Appendix 3 [file publichealth_v3i4e64_app3.pdf]

Table 1. Mean rank of the daily Baidu Index for the term "lung cancer"(B), the daily Baidu media index (M)and the daily PM2.5 concentration (P) in different months form 2014 to2016.

city	Month	N	B	P	M
Beijing	Jan	93	616.63	590.78	517.72
	Feb	85	580.3	558.59	509.15
	Mar	93	670.25	586.37	542.88
	Apr	90	581.16	591.06	650.53
	May	93	632.86	490.31	558.76
	Jun	90	454.31	473.63	663.74
	Jul	93	354.95	568.32	478.34
	Aug	93	366.17	429.8	422.66
	Sep	90	432.79	449.28	452.52
	Oct	93	483.18	568.62	459.04
	Nov	90	602.1	648.56	705.18
	Dec	93	806.04	626.53	627.04
Changchun	Jan	93	613.73	818.98	517.72
	Feb	85	594.51	668.18	509.15
	Mar	93	706.01	644.67	542.88
	Apr	90	614.44	537.47	650.53
	May	93	570.55	345.15	558.76
	Jun	90	562.63	374.98	663.74
	Jul	93	527.32	398.73	478.34
	Aug	93	437.99	301.63	422.66
	Sep	90	444.79	262.91	452.52
	Oct	93	446.91	680.11	459.04
	Nov	90	536.38	756.4	705.18
	Dec	93	529.55	794.63	627.04
Changsha	Jan	93	688.41	892.77	517.72
	Feb	85	593.2	700.54	509.15
	Mar	93	673.84	610.31	542.88
	Apr	90	702.76	489.07	650.53
	May	93	700.47	506.31	558.76
	Jun	90	562.92	364.38	663.74
	Jul	93	392.71	320.11	478.34
	Aug	93	413.53	350.2	422.66
	Sep	90	442.46	496.77	452.52
	Oct	93	446.69	618.64	459.04
	Nov	90	465.09	511.74	705.18
	Dec	93	503.09	723.52	627.04
Chengdu	Jan	93	446.41	863.88	517.72
	Feb	85	430.46	728.12	509.15
	Mar	93	521.13	678.98	542.88

	Apr	90	529.44	541.17	650.53
	May	93	522.08	556.14	558.76
	Jun	90	505.01	350.06	663.74
	Jul	93	500.04	314.02	478.34
	Aug	93	497.77	350.92	422.66
	Sep	90	624.15	331.23	452.52
	Oct	93	657.09	512.31	459.04
	Nov	90	649.58	567.11	705.18
	Dec	93	692.37	790.47	627.04
Chongqing	Jan	93	619.4	880.09	517.72
	Feb	85	635.75	742.36	509.15
	Mar	93	699.61	573.33	542.88
	Apr	90	529.99	450.02	650.53
	May	93	628.7	468.37	558.76
	Jun	90	487.41	356.57	663.74
	Jul	93	367.68	339.67	478.34
	Aug	93	447.47	373.4	422.66
	Sep	90	478.99	472.53	452.52
	Oct	93	515.54	529.49	459.04
	Nov	90	544.51	624.07	705.18
	Dec	93	629.52	779.38	627.04
Fuzhou	Jan	88	568.74	772.41	521.06
	Feb	85	560.78	617.22	506.08
	Mar	93	710.69	726.98	539.77
	Apr	90	700.58	660.86	646.96
	May	93	696.77	620.77	555.57
	Jun	90	595.39	406.68	660.1
	Jul	93	495.8	334.03	475.57
	Aug	93	468.64	385.68	420.05
	Sep	90	415.02	384.17	449.74
	Oct	93	470.91	457.57	456.22
	Nov	90	428.29	512.22	701.33
	Dec	93	441.45	684.59	623.61
Guangzhou	Jan	93	627.74	751.69	517.72
	Feb	85	488.69	617.69	509.15
	Mar	93	748.46	619.89	542.88
	Apr	90	746.81	603.47	650.53
	May	93	755.39	389.1	558.76
	Jun	90	586.59	282.48	663.74
	Jul	93	452.87	365.38	478.34
	Aug	93	396.28	503.79	422.66
	Sep	90	428.28	538.29	452.52
	Oct	93	477.55	630.17	459.04

	Nov	90	442.64	600.35	705.18
	Dec	93	425.87	680.18	627.04
Guiyang	Jan	93	598.53	839.95	517.72
	Feb	85	556.45	740.96	509.15
	Mar	93	731.32	648.35	542.88
	Apr	90	550.69	596.06	650.53
	May	93	632.59	493.61	558.76
	Jun	90	510.33	319.11	663.74
	Jul	93	472.24	253.25	478.34
	Aug	93	513.67	353.23	422.66
	Sep	90	459.3	459.12	452.52
	Oct	93	452.76	604.18	459.04
	Nov	90	552.51	514.13	705.18
	Dec	93	548.39	766.76	627.04
Haerbin	Jan	93	653.33	873.74	517.72
	Feb	85	490.98	789.45	509.15
	Mar	93	711.92	620.59	542.88
	Apr	90	620.77	545.58	650.53
	May	93	578.45	311.75	558.76
	Jun	90	521.32	320.07	663.74
	Jul	93	363.53	356.03	478.34
	Aug	93	430.92	274.87	422.66
	Sep	90	398.43	228.76	452.52
	Oct	93	463.6	584.94	459.04
	Nov	90	642.33	847.32	705.18
	Dec	93	701.1	841.49	627.04
Haikou	Jan	93	599.02	839.4	517.72
	Feb	85	618.38	746.51	509.15
	Mar	93	646.69	607.35	542.88
	Apr	90	636.23	617.84	650.53
	May	93	628.61	417.83	558.76
	Jun	90	525.17	254.23	663.74
	Jul	93	419.37	284.85	478.34
	Aug	93	477.26	332.17	422.66
	Sep	90	530.14	453.51	452.52
	Oct	93	495.63	725.32	459.04
	Nov	90	521.77	516.59	705.18
	Dec	93	490.37	792.06	627.04
Hangzhou	Jan	93	653.7	819.31	517.72
	Feb	85	600.78	635.08	509.15
	Mar	93	721.54	641.15	542.88
	Apr	90	719.22	570.56	650.53
	May	93	710.79	556.91	558.76

	Jun	90	449.63	421.42	663.74
	Jul	93	376.05	315.2	478.34
	Aug	93	386.36	330.7	422.66
	Sep	90	490.75	402.27	452.52
	Oct	93	462.38	493.97	459.04
	Nov	90	494.31	617.35	705.18
	Dec	93	519.68	779.63	627.04
Hefei	Jan	93	641.85	836.31	517.72
	Feb	85	696.66	672.25	509.15
	Mar	93	706.27	673.27	542.88
	Apr	90	711.73	516.13	650.53
	May	93	757.7	536.65	558.76
	Jun	90	535.77	473.33	663.74
	Jul	93	468.47	344.29	478.34
	Aug	93	412.04	326.13	422.66
	Sep	90	394.59	369.15	452.52
	Oct	93	421.9	491.48	459.04
	Nov	90	378.41	601.75	705.18
	Dec	93	463.75	744.37	627.04
Huhehot	Jan	93	616.41	740.22	517.72
	Feb	85	603.8	629.22	509.15
	Mar	93	668.47	597.49	542.88
	Apr	90	613.73	488.64	650.53
	May	93	592.17	489.15	558.76
	Jun	90	479.62	346.59	663.74
	Jul	93	443.53	411.94	478.34
	Aug	93	520.46	306.11	422.66
	Sep	90	509.34	394.23	452.52
	Oct	93	451.61	596.26	459.04
	Nov	90	539.62	762.23	705.18
	Dec	93	546.32	820.33	627.04
Jinan	Jan	93	695.27	773.25	517.72
	Feb	85	725.65	640.5	509.15
	Mar	93	783.59	583.54	542.88
	Apr	90	689.66	573.91	650.53
	May	93	681.11	437.7	558.76
	Jun	90	520.51	443.93	663.74
	Jul	93	415.48	459.73	478.34
	Aug	93	413.4	346.23	422.66
	Sep	90	311.97	468.23	452.52
	Oct	93	326.11	500.77	459.04
	Nov	90	414.32	625.88	705.18
	Dec	93	611.87	733.59	627.04

Kunming	Jan	93	551.09	701.38	517.72
	Feb	85	510.59	646.56	509.15
	Mar	93	601.72	782.8	542.88
	Apr	90	623.98	761.11	650.53
	May	93	606.94	546.9	558.76
	Jun	90	494.74	244.34	663.74
	Jul	93	542.81	359.89	478.34
	Aug	93	594.77	346.19	422.66
	Sep	90	589.53	424.21	452.52
	Oct	93	466.03	581.65	459.04
	Nov	90	524.22	480.54	705.18
	Dec	93	473.55	705.71	627.04
Lanzhou	Jan	93	647.98	730.23	517.72
	Feb	85	578.61	671.84	509.15
	Mar	93	635.83	564.65	542.88
	Apr	90	544.55	445.91	650.53
	May	93	533.89	484.65	558.76
	Jun	90	491.45	434.78	663.74
	Jul	93	486.95	388.37	478.34
	Aug	93	516.98	330.9	422.66
	Sep	90	494.12	397.88	452.52
	Oct	93	492.94	486.47	459.04
	Nov	90	549.44	783.61	705.18
	Dec	93	608.17	869.1	627.04
Lasa	Jan	93	514.63	600.7	517.72
	Feb	85	472.16	487.98	509.15
	Mar	93	521.84	449.33	542.88
	Apr	90	551.07	688.14	650.53
	May	93	588.56	609.83	558.76
	Jun	90	565.17	397.56	663.74
	Jul	93	592.81	320.89	478.34
	Aug	93	611.08	330.61	422.66
	Sep	90	504.72	273.9	452.52
	Oct	93	527	604.01	459.04
	Nov	90	521.62	901.71	705.18
	Dec	93	603.11	914.31	627.04
Nanchang	Jan	93	598.84	823.82	517.72
	Feb	85	643.18	633.48	509.15
	Mar	93	756.63	619.44	542.88
	Apr	90	657.02	481.94	650.53
	May	93	640.62	556.39	558.76
	Jun	90	473.11	356.02	663.74
	Jul	93	413.35	331.49	478.34

	Aug	93	372.25	348.23	422.66
	Sep	90	467.79	480.75	452.52
	Oct	93	453.87	638.65	459.04
	Nov	90	546.65	550.57	705.18
	Dec	93	565.23	758.06	627.04
Nanjing	Jan	93	616.84	812.62	517.72
	Feb	85	564.8	692.08	509.15
	Mar	93	638.32	686.34	542.88
	Apr	90	630.46	541.16	650.53
	May	93	687.44	585.63	558.76
	Jun	90	567	540.87	663.74
	Jul	93	428.96	420.41	478.34
	Aug	93	417.05	307.84	422.66
	Sep	90	480.47	365.3	452.52
	Oct	93	490.69	353.51	459.04
	Nov	90	492.76	585.3	705.18
	Dec	93	567.88	698.08	627.04
Nanning	Jan	93	592.56	824.53	517.72
	Feb	85	550.35	761.82	509.15
	Mar	93	688.74	661.74	542.88
	Apr	90	676.65	610.17	650.53
	May	93	645.27	417.19	558.76
	Jun	90	582.82	245.38	663.74
	Jul	93	536.77	279.38	478.34
	Aug	93	455.16	354.23	422.66
	Sep	90	527.68	469.57	452.52
	Oct	93	427.04	702.66	459.04
	Nov	90	447.82	517.94	705.18
	Dec	93	452.6	744.42	627.04
Shanghai	Jan	93	531.14	724.04	517.72
	Feb	85	544.81	607.17	509.15
	Mar	93	662.74	610.96	542.88
	Apr	90	567.34	662.71	650.53
	May	93	570.67	618.38	558.76
	Jun	90	429.99	496.1	663.74
	Jul	93	460.24	463.85	478.34
	Aug	93	535.6	338.96	422.66
	Sep	90	522.72	366.82	452.52
	Oct	93	560.59	397.35	459.04
	Nov	90	573.91	571.51	705.18
	Dec	93	618.7	726.08	627.04
Shenyang	Jan	93	597.08	754.86	513.18
	Feb	85	606.74	667.15	504.86

	Mar	93	627.81	651.69	538.32
	Apr	84	580.9	561.8	672.57
	May	93	708.06	402.19	554.18
	Jun	90	489.48	368.74	658.89
	Jul	93	450.32	385.37	474.34
	Aug	93	493.92	309.75	418.63
	Sep	90	420.68	349.69	448.35
	Oct	93	421.79	592.62	454.95
	Nov	90	573.13	778.5	699.92
	Dec	93	579.84	731.19	622.11
Shijiazhuang	Jan	93	702.67	783.61	517.72
	Feb	85	682.71	635.26	509.15
	Mar	93	702.58	601.69	542.88
	Apr	90	676.7	568.7	650.53
	May	93	741.6	405.2	558.76
	Jun	90	506.04	407.57	663.74
	Jul	93	402.76	479.12	478.34
	Aug	93	368.59	325.64	422.66
	Sep	90	388.72	388.17	452.52
	Oct	93	370.16	539.99	459.04
	Nov	90	456.81	682.08	705.18
	Dec	93	588.87	767.66	627.04
Taiyuan	Jan	93	697.32	669.27	517.72
	Feb	85	647.19	559.26	509.15
	Mar	93	576.73	562.53	542.88
	Apr	90	636.51	515.87	650.53
	May	93	672.61	477.96	558.76
	Jun	90	493.97	435.89	663.74
	Jul	93	472.73	432.76	478.34
	Aug	93	412.48	344.99	422.66
	Sep	90	447.75	416.94	452.52
	Oct	93	437.43	624.08	459.04
	Nov	90	484.54	766.88	705.18
	Dec	93	607	774.62	627.04
Tianjin	Jan	93	644.53	644.59	517.72
	Feb	85	701.44	529.44	509.15
	Mar	93	667.18	665.88	542.88
	Apr	90	569.42	585.17	650.53
	May	93	618.71	475.65	558.76
	Jun	90	469.26	474.47	663.74
	Jul	93	390.26	494.05	478.34
	Aug	93	401.65	400.61	422.66
	Sep	90	412.84	361.58	452.52

	Oct	93	420.42	519.23	459.04
	Nov	90	603.03	694.28	705.18
	Dec	93	691.92	732.89	627.04
Urumqi	Jan	93	650.16	918.39	517.72
	Feb	85	625.63	845.41	509.15
	Mar	93	598.03	716.09	542.88
	Apr	90	551.98	413.95	650.53
	May	93	586.98	373.74	558.76
	Jun	90	494.94	307.6	663.74
	Jul	93	479.38	292.99	478.34
	Aug	93	469.83	314.04	422.66
	Sep	90	497.78	350.54	452.52
	Oct	93	451.61	512.72	459.04
	Nov	90	583.15	644.67	705.18
	Dec	93	597.04	901.99	627.04
Wuhan	Jan	93	567.31	925.95	517.72
	Feb	85	571.33	751.32	509.15
	Mar	93	729.66	693.3	542.88
	Apr	90	622.99	486.77	650.53
	May	93	627.78	518.78	558.76
	Jun	90	477.58	395.86	663.74
	Jul	93	388.17	242.86	478.34
	Aug	93	405.85	331.41	422.66
	Sep	90	484.21	374.68	452.52
	Oct	93	522.15	537.8	459.04
	Nov	90	521.51	556.46	705.18
	Dec	93	662.58	772	627.04
Xian	Jan	93	681.93	803.68	517.72
	Feb	85	617.35	728.71	509.15
	Mar	93	622.08	689.61	542.88
	Apr	90	665.56	521.68	650.53
	May	93	806.47	440.87	558.76
	Jun	90	583.42	317.6	663.74
	Jul	93	456.58	276.45	478.34
	Aug	93	413.34	363.68	422.66
	Sep	90	355.21	401.18	452.52
	Oct	93	361.6	581.66	459.04
	Nov	90	467.19	685.74	705.18
	Dec	93	553.26	778	627.04
Xining	Jan	93	599.55	786.65	517.72
	Feb	85	529.77	726.54	509.15
	Mar	93	619.11	670.62	542.88
	Apr	90	528.3	507.86	650.53

	May	93	615.7	453.74	558.76
	Jun	90	499.49	358.49	663.74
	Jul	93	529.45	310.85	478.34
	Aug	93	557.97	369.57	422.66
	Sep	90	478.09	331.74	452.52
	Oct	93	491.52	442.03	459.04
	Nov	90	515.93	780.01	705.18
	Dec	93	609.95	852.26	627.04
Yinchuan	Jan	93	643.62	778.28	517.72
	Feb	85	575.82	697.32	509.15
	Mar	93	632.42	603.34	542.88
	Apr	90	588.73	538.69	650.53
	May	93	591.56	385.46	558.76
	Jun	90	515.18	336.67	663.74
	Jul	93	516.33	421.07	478.34
	Aug	93	514.94	421.04	422.66
	Sep	90	537.53	344.07	452.52
	Oct	93	436.93	518.73	459.04
	Nov	90	515.86	780.73	705.18
	Dec	93	514.25	763.15	627.04
Zhengzhou	Jan	93	642.95	803.25	517.72
	Feb	85	689.84	694.05	509.15
	Mar	93	756.08	646.1	542.88
	Apr	90	688.51	571.88	650.53
	May	93	725.24	480.33	558.76
	Jun	90	483.48	442.21	663.74
	Jul	93	396.53	316.56	478.34
	Aug	93	394.75	327.11	422.66
	Sep	90	401.14	417.64	452.52
	Oct	93	405.46	481.75	459.04
	Nov	90	449.01	685.63	705.18
	Dec	93	555.65	725.53	627.04
